# Cyberbullying and Psychological Well-being in Young Adolescence: The Potential Protective Mediation Effects of Social Support from Family, Friends, and Teachers

**DOI:** 10.3390/ijerph17010045

**Published:** 2019-12-19

**Authors:** Karin Hellfeldt, Laura López-Romero, Henrik Andershed

**Affiliations:** 1 School of Law, Psychology and Social Work, Örebro University, SE-701 82 Örebro, Sweden; henrik.andershed@oru.se; 2 Department of Clinical Psychology and Psychobiology, Universidade de Santiago de Compostela, 15782 Santiago de Compostela, Spain; laura.lopez.romero@usc.es

**Keywords:** cyberbullying, adolescents, cyber-victim, cyberbully-victim, mental health, psychological well-being, social support, depression, anxiety, subjective well-being

## Abstract

In the current study, we tested the relations between cyberbullying roles and several psychological well-being outcomes, as well as the potential mediation effect of perceived social support from family, friends, and teachers in school. This was investigated in a cross-sectional sample of 1707 young adolescents (47.5% girls, aged 10–13 years, self-reporting via a web questionnaire) attending community and private schools in a mid-sized municipality in Sweden. We concluded from our results that the Cyberbully-victim group has the highest levels of depressive symptoms, and the lowest of subjective well-being and family support. We also observed higher levels of anxiety symptoms in both the Cyber-victims and the Cyberbully-victims. Moreover, we conclude that some types of social support seem protective in the way that it mediates the relationship between cyberbullying and psychological well-being. More specifically, perceived social support from family and from teachers reduce the probability of depressive and anxiety symptoms, and higher levels of social support from the family increase the probability of higher levels of subjective well-being among youths being a victim of cyberbullying (i.e., cyber-victim) and being both a perpetrator and a victim of cyber bullying (i.e., cyberbully-victim). Potential implications for prevention strategies are discussed.

## 1. Introduction

The negative consequences of school bullying have been relatively well established within research [1,2,3]. However, due to technology development, bullying is not only restricted to the physical and real-life school context. Cyberbullying refers to an intentional act of aggression, carried out to harm another individual using electronic forms of contacts or devices [4]. Previous studies have rather consistently linked cyberbullying with several negative psychosocial well-being outcomes in adolescence (for review, see Reference [5]). However, much more research is needed to more clearly establish how and to what extent the distinctive cyberbullying roles (i.e., being a cyberbully, cyber-victim, and cyberbully-victim) are related with various psychological well-being outcomes. More research investigating if and to what extent various types of social support can mediate these relations is also needed. Social support from family, friends, and teachers has proven to be able to mitigate the negative impacts of traditional bullying but have been scarcely studied in relation to cyberbullying [6,7,8,9,10,11]. Therefore, the aim of the present study is to examine the relations between cyberbullying and its different roles with several psychological well-being outcomes. In addition, and in order to identify potential variables that may influence these associations, we examine if and to what extent perceived social support from the family, friends, and teachers mediate the association between cyberbullying roles and psychological well-being outcomes.

### 1.1. The Association between Cyberbullying and Adolescent Psychological Well-Being

Some scholars suggest that cyberbullying is more stressful than traditional forms of school bullying [12,13]. Cyberbullying has emerged as a distinct form of bullying, with features such as publicity, permeability of online messages and pictures, anonymity of offender, and limitless boundaries, which distinguish it from traditional school bullying [4]. Hence, it is possible that cyberbullying renders other and more serious consequences as compared to traditional bullying. Although less studied than traditional bullying, involvement in cyberbullying has been linked to a range of psychological problems (for review, see Reference [5]). However, very few studies have compared psychological outcomes for the different cyberbullying roles. Youths may participate in cyberbullying either in the role of cyber victims, cyberbullies, or cyberbully-victims. Previous research has mostly focused on victims of cyberbullying and its potential consequences. A substantial amount of research has found associations between cyber-victimization and depressive symptoms ([13,14,15,16]; for reviews and meta-analyses, see Reference [17]). Fewer studies have studied other outcomes and found relations between cyber-victimization and anxiety symptoms [18] and between cyber-victimization and lower levels of subjective well-being [19,20].

Although less studied, research also indicates an association between cyberbullying (i.e., being in the role of the perpetrator of cyberbullying) and adverse outcomes. A majority of studies show that higher levels of cyberbullying relates to higher levels of depressive symptoms [14,18], anxiety symptoms [18,21], and lower levels of subjective well-being [19]. However, some studies indicate that cyberbullies might be better off than those who are victimized, also with examples of studies finding no relation between the role of cyberbullying and depressive symptoms [15]. 

Some studies suggest that children and adolescents who are both victims and perpetrators of cyberbullying, (i.e., cyberbully-victims), constitute a distinct group with the highest risk for psychosocial problems, such as depressive and anxiety symptoms, as well as for lower levels of well-being in general [22,23,24,25]. Nevertheless, a limited amount of research has focused on the relationship between cyberbully-victims and different well-being outcomes. 

In sum, existing research suggests that children involved in any way in cyberbullying can be at increased risk for psychological distress, including depressive and anxiety symptoms, as well as lower subjective well-being. Existing studies are, however, not entirely consistent in these findings and more research is therefore clearly needed. 

### 1.2. The Potential Positive and Protective Role of Social Support 

Very little is known from research concerning how to prevent or ameliorate the potential negative consequences of cyberbullying. One such potential protective factor that could mediate the relationship between cyberbullying and negative well-being could be social support. Existing studies indicate that different types of social support could potentially buffer against negative consequences of traditional, non-cyber, bullying [6,7,8,9,10,11]. The term social support often refers to different kinds of supportive social relations or interactions that can increase or promote an individuals’ well-being by acting as a buffering factor against negative outcomes [26,27,28]. Social support can be defined as including both an emotional dimension, (i.e., the individuals’ perception of being valued and cared for by others in their social network), as well as an instrumental dimension, (i.e., the individuals’ perception of having access to practical help with different tasks or obstacles in life) [29,30]. Theoretically, the potential benefits of social support can be understood in terms of the stress-buffering model [26], that is, that social support works as a buffer in stressful situations by serving as an important coping mechanism on which youths can draw [31]. Ongoing involvement in cyberbullying can be seen as a chronic stressor. Hence, emotional and instrumental social support could serve as important resources when youths experience bullying, by both offering support when it occurs, but also to help stop bullying at an early stage [9]. For example, greater family support has been shown to be able to protect adolescents from being cyberbullied and cyber-victimized [32]. In addition, telling a friend about the bullying situation has been identified by adolescents themselves as the most helpful coping strategy when being cyberbullied [33]. 

Social support may derive from a number of sources and can thus be of various types. Among youths, the two primary sources or types of support seem to be parents and friends [7]. However, which of these two types of social support that serves as the primary resource seem to differ with age during youth. Younger children receive their primary support from parents but as the child approaches and enters adolescence, the role of parents can become less prominent, and the support from friends can increase and become more important [34]. Turning to a friend for support has been shown to be more commonly used by cyberbullied children, as compared to other types of support [35]. Regarding youths’ social network in the context of the school, teachers could be expected to play an important role in offering support [36]. Although cyberbullying generally takes place outside the school, it has been shown that most of the victims know their perpetrator from school [37]. Hence, teachers can potentially serve as an important support system when children experience cyberbullying. Importantly, seeking support from parents, friends, or teachers have been shown to be quite common strategies used by adolescents to cope with cyberbullying experiences [35]. 

Although there are several studies on different programs aimed at preventing bullying, few studies have focused on factors that may help youths to handle cyberbullying [38]. Social support from parents, friends, or teachers has been indicated to be able to mitigate the consequence of being a victim of traditional bullying but studies concerning this topic have yielded mixed findings [6,9,10]. In one study, moderate levels of peer support were shown to buffer against anxiety/depression among bullies and victims, as well as bully-victims [7]. In contrast, others found that support from friends and family protected cyber-victims from poor academic achievement but not from mental health difficulties [9]. Also, little research has explicitly investigated various types of social support in relation to cyber-victimization in general, and different roles of cyberbullying more specifically. An exception is a study including 765 Swiss seventh-graders, which examined if certain coping strategies could moderate the relation between cyber-victimization and depressive symptoms [39]. In this study, seeking support from friends and family showed a significant buffering effect on depressive symptoms. In addition, in a study including 1416 adolescents living in Cyprus, family support protected both cyber-victims and cyberbullies from being cyber-victimized one year later [32]. These previous studies offer promising indications that social support can protect adolescents involved in cyberbullying from negative consequences. However, little empirical work has been done in this area and more research is needed to understand how the various cyberbullying roles among youths can benefit from different types of social support. 

### 1.3. The Present Study 

Even though more and more research has linked cyberbullying to different aspects of psychological distress, there is still a lack of studies focused on how distinctive cyberbullying roles (i.e., being a cyberbully, cyber-victim, and cyberbully-victim) are associated with various adverse outcomes [22]. Furthermore, few studies have examined processes that could mitigate the potential negative consequences of cyberbullying. Previous studies suggest that social support from family, friends, and teachers may mitigate the effect of traditional bullying [6] but this has not been thoroughly studied in relation to cyberbullying in general, nor more specifically in relation to distinctive cyberbullying roles. 

Hence, in the current study, we examine the relations between cyberbullying and its different roles with symptoms of depression, anxiety, and levels of subjective well-being. In addition, and in order to identify potential variables that may mediate these associations, we test whether and to what extent youths’ perceived social support from the family, friends, and teachers mediate the association between cyberbullying roles and the three studied psychological well-being outcomes. This was examined using data from a cross-sectional study, in which 1707 youths aged 10–13 years in a mid-sized Swedish municipality responded to a questionnaire. The specific research questions we aimed to answer are:

- How are different cyberbullying roles (i.e., being a cyberbully, cyber-victim, and cyberbully-victim) associated with depressive and anxiety symptoms, as well as with subjective well-being?
- To what extent can youths´ perceived social support from family, friends, and teachers mediate the association between various cyberbullying roles and depressive and anxiety symptoms, as well as to subjective well-being?

In terms of hypotheses, previous research indicates a negative influence of cyberbullying on children’s psychological well-being [5,19]. Hence, we hypothesized higher levels of depressive and anxiety symptoms, and lower levels of subjective well-being for the cyber-victim and cyberbully-victim roles. We also expected protective mediation effects of social support in the way that social support from family, friends, and teachers will reduce the risk for depressive and anxiety symptoms and lower the levels of subjective well-being among those exposed to cyberbullying.

We also controlled for gender since prior research indicates that seeking support may be more beneficial for girls compared to boys [9], and also that different sources of support might be of different importance for bullied girls and boys [27].

## 2. Materials and Methods 

This study uses cross-sectional self-report data from the fifth wave of data collections in an ongoing prospective longitudinal study, the SOFIA-study (Social and Physical Development, Interventions and Adaptation). The SOFIA-study aims to provide better understanding of children’s behavior, social adjustment, and psychological and physical health. The target population for the SOFIA-study was all children born in 2005, 2006, and 2007, attending preschools during the spring of 2010 (2542 children) in a midsized (approximately 85,000 citizens) Swedish municipality. In terms of demographics of the municipality (i.e., gender, age, educational level, and employment, and the mix of urban and rural areas), the municipality is proportional to the rest of Sweden. Wave 5 of the SOFIA-study used in the current study was conducted in the year 2018 when the children were in their young adolescent age of 10–13 years. In the first data collection, the parents of 2121 children (85.7% of target population; 47% girls) gave active consent to participate. The SOFIA-study uses parents’, teachers’, and (pre)schoolteachers’ reports for all five waves of data collections. In the fifth data collection, the children were themselves the respondents for the first time. In Wave 5, 1707 children (approximately 80% of the original sample) completed the self-report questionnaire. The current study used these cross-sectional youth self-reports from the fifth wave of data collection of the SOFIA-study.

### 2.1. Participants 

The sample consisted of 1707 youths, which represent 80.4% of the original target sample (47.5% girls). The sample age ranged between 10 to 13 years (*Mean*_age_ = 11.89, *Standard Deviation*_age_ = 0.86) and included 33.7% (n = 576) children born in 2005, 33.7% (n = 576) children born in 2006, and 32.5% (n = 554) children born in 2007. In addition, 16.7% of participants had at least one parent born in another country than Sweden. 

Regarding non-participants, caregivers declined participation in the study for about 16% of the target population in 2010. The non-participants did not differ significantly from participants regarding relevant aspects, such as the children’s levels of conduct problems and internalizing problems, or the caregivers’ socio-economic status and origin [40].

### 2.2. Measurements 

Cyberbullying/Cyber-victimization. Two items from the Revised Olweus’ Bully/Victim Questionnaire (OBVQ, [41]) were used to assess cyberbullying. Each participant was introduced with a detailed definition of bullying. The definition included three common criteria of bullying, i.e., intentionality, repetitiveness, and power imbalance between perpetrator(s) and a victim [41,42]. This definition was followed up by two cyberbullying questions, one on cyber-victimization, “How often have you been cyber-victimized during the past six months?”, and one on cyberbullying, “How often have you cyberbullied other students at school during the past six months?”. Items were rated as 1 (Never), 2 (1 or 2 times), 3 (2 or 3 times a month), 4 (Once a week), and 5 (Several times a week). The cyberbullying questions were preceded by the following definition: “Here are some questions about cyberbullying. When we say ‘cyberbullying’, we mean bullying through e-mail, instant messaging, in a chat room, on a website, or through a text message sent to a cell phone”. Following recommendations from previous studies, a cutoff point for frequent involvement in bullying is “two or three times a month”, and “only once or twice” for occasional involvement [43]. 

Depressive symptoms. To assess Depressive symptoms, items from The Youth Self-Report (YSR) was used, an instrument based on the Achenbach System of Empirically Based Assessment [44]. The subscale used in this study, i.e., Affective problems (used to measure depressive symptoms) reflects DSM-IV (Diagnostic and Statistical Manual of Mental Disorders, 4th) problem dimensions, which comprise items that experienced psychiatrists and psychologists from 16 cultures have rated as being very consistent with DSM-IV diagnostic categories [45]. We included all items from the Affective problems subscale but one (i.e., “Thinks about suicide”), resulting in 11 items (e.g., “I am unhappy, sad, or depressed”; *α* = 0.79; mean inter item correlation (MIC) = 0.44). Participants rated each of the 11 included items on a three-point scale ranging from 1 (Not true) to 3 (Very true or often true). Respondents were requested to base their ratings on the preceding 6 months. 

Anxiety Symptoms. Anxiety symptoms were assessed with items from the Spence Children´s Anxiety Scale (SCAS [46]). The SCAS corresponds to DSM-IV anxiety disorder categories, and the scale has shown good psychometric properties with empirical support for test–retest reliability and internal consistency [47]. In the current study, we used the subscale for general anxiety, in total 6 items (e.g., “I worry that something awful will happen to me”; *α* = 0.81, MIC = 0.58). Participants rated each item on a four-point scale ranging from 1 (Never) to 4 (Always). Respondents were requested to base their ratings on the preceding 6 months. 

Subjective well-being. Subjective well-being was assessed using one item (“I enjoy life very much”), using a response scale ranging from 1 (Does not apply at all) to 4 (Applies very well). Respondents were requested to base their ratings on the preceding 6 months.

Perceived social support. Social support was assessed with The Multidimensional Scale of Perceived Social Support [48]. This scale consists of 12 items intended to assess perceived social support from Family (four items; e.g., “My family is willing to help me making decisions”; *α* = 0.81, MIC = 0.63), Friends (four items, e.g., “I can talk about my problems with my friends”; *α* = 0.87, MIC = 0.72), and Teachers (four items; e.g., “My teachers help me solve problems in a good way”; *α* = 0.91, MIC = 0.80). Participants rated each item in a response scale ranging from 1 (Does not apply at all) to 4 (Applies very well). Respondents were requested to base their ratings on the preceding 6 months.

### 2.3. Procedure

Initially, the decision makers in the Child and Adolescent Department at the municipality, decided on the participation of all municipal preschools. Private preschool principals were contacted separately. In 2010, at the first wave of data collection, all concerned preschool teachers received written information about the study, who in turn, passed on this information to parents who gave active informed consent. In the fifth wave of data collection carried out in the year of 2018 (used in current study), the youths answered a web-based questionnaire during school hours. They did not receive any compensation for their participation. The web-questionnaire took approximately 20 minutes to complete. Each school was responsible for administrating the questionnaire, i.e., giving the children the possibility to answer the questionnaire via a secure web-based questionnaire. The youths received information about the purpose of the study, that their participation was voluntary, etc. 

The SOFIA-study has been evaluated by a regional ethics committee (First three waves 2010–2012; Dnr #2009/429. Fourth wave 2015; Dnr #2015/024. Fifth wave 2018; Dnr #2017/486). The study has followed all stipulated ethical research principles by the Swedish Research Council and the Swedish Ethics Authority.

### 2.4. Statistical Analyses 

First, descriptive statistics were computed for all study variables, with additional tests for differences due to gender and age performed through Student’s *t*-test and zero-order correlations, respectively. Second, correlations among study variables were explored with zero-order and partial correlations, controlling for gender and age. Third, cyberbullying role variables (i.e., No cyberbully/cyber-victim, Cyberbully, Cyber-victim, Cyberbully-victim) were created by computing low/high groups from the cyberbullying and cyber-victimization items. These groups were then compared on psychological well-being outcomes (i.e., depressive symptoms, anxiety symptoms, and subjective well-being) and perceived social support (i.e., social support from family, friends, and teachers) through analysis of variance (ANOVA) and including the Bonferroni correction for multiple comparisons (*p* < 0.008). The strength of differences was assessed with the partial effect size statistic (*ƞ²*), and interpreted as small (>0.05), medium (0.06 to 0.14), and large (<0.14). Both correlation analyses and comparisons across groups were replicated using non-parametric analytic approaches (i.e., Spearman rho and Kruskal–Wallis tests, respectively), with no relevant changes in main results (further information is available upon request). Descriptive statistics, correlation analyses, and ANOVAs were computed in IBM SPSS 20 (IBM, Armonk, NY, USA). 

Finally, a series of mediation models were examined via structural equation modeling (SEM) in Mplus 7.4 (Muthén & Muthén, Los Angeles, CA, USA). The estimated models tested the effects of cyberbullying roles (i.e., Cyberbully, Cyber-victim, and Cyberbully-victim), coded as dummy variables (0 = No, 1 = Yes), on depressive and anxiety symptoms, and subjective well-being through the potential mediation effect of perceived family, friends, and teachers support, controlling for age and gender. The mean- and variance-adjusted maximum likelihood test statistic (MLMV) was used as the estimator, since it is robust to non-normal data, and yields the best combination of accurate standard errors and Type I error [49]. Goodness-of-fit was assessed using the root-mean-square error of approximation (RMSEA), the comparative fit index (CFI), and the standardized root mean square residual (SRMR). According to suggestions by Hu and Bentler [50], RMSEA and SRMR values lower or equal to 0.06 and 0.05 respectively, and CFI values of 0.95 or higher are considered indicators of good model fit, whereas a RMSEA and SRMR smaller than 0.08, and CFI larger than 0.90 indicate adequate model fit.

## 3. Results

### 3.1. Descriptive Statistics and Correlations between Main Study Variables

Descriptive statistics and gender comparisons are presented in Table 1. As expected given the characteristics of the sample, participants showed low levels of cyberbullying and cyber-victimization, depression, and anxiety symptoms, and high levels of subjective well-being, as well as high levels of perceived social support. Gender comparisons revealed differences across gender groups in cyberbullying, anxiety, subjective well-being, and peer support, with effect sizes ranging from low to moderate. Results showed higher levels of cyberbullying and subjective well-being among boys, and higher levels of anxiety and social support from friends among girls. Significant differences were also observed in terms of age for all study variables (r_s_ = 0.05 to −0.26; *p* < 0.05). More specifically, results revealed higher levels of cyberbullying and cyber-victimization, depressive, and anxiety symptoms among the older adolescents, whereas higher levels of subjective well-being and perceived social support were observed among the younger adolescents. 

Zero-order correlation results between the main study variables are displayed in Table 2. All variables were significantly correlated with each other, except cyberbullying and anxiety symptoms. Results from partial correlations controlling for age and gender yielded basically the same results, with similar values for all the analyzed variables. The only larger difference was observed for the correlation between cyberbullying and friends’ support, which was no longer significant when controlling for age and gender (results available upon request). 

### 3.2. Cyberbullying Roles: Comparisons across Groups on Psychological Well-Being

In order to examine the associations between distinctive cyberbullying roles and psychological well-being, we identified groups low and high from the cyberbullying and cyber-victimization continuous items. Based on prior recommendations, those participants who reported being cyberbullying/bullied “two or three times a month” or more often [42] were classified as cyberbullies (n = 11; 0.6% of the sample) and cyber-victims (n = 55; 3.2%), respectively. By combining these groups, four mutually exclusive groups representing distinctive cyberbullying roles were identified: No Cyberbully/cyber-victim (n = 1640; 96.5%), Cyberbully (n = 5; 0.3%), Cyber-victim (n = 49; 2.8%), and Cyberbully-victim (n = 6; 0.4%). Given the low prevalence rates in the Cyberbully, Cyber-victim, and Cyberbully-victim groups, and in order to ensure enough participants within each group for subsequent analyses, new cyberbullying groups were created using a less stringent cut-off. The less strict cutoff follows recommendations from previous studies, resulting in groups with youths more occasionally involved in cyberbullying [43]. Hence, those participants who reported being cyberbullying/bullied “one or two times” or more often were classified as cyberbullies (n = 42; 2.5%) and cyber-victims (n = 218; 12.8%). The combination of these groups yielded four different groups representing the following cyberbullying roles: No Cyberbully/cyber-victim (n = 1469; 86.4%), Cyberbully (n = 13; 0.8%), Cyber-victim (n = 191; 11.2%), and Cyberbully-victim (n = 27; 1.6%). Comparisons across groups revealed no differences in terms of gender, χ² (3) = 7.39; *p* = 0.060, nor age, *F* = 1.73; *p* = 0.158 in the distribution across these groups. 

Results of comparisons between these groups showed significant differences on all three of the psychological well-being outcomes as well as on all types of perceived social support, even after applying the Bonferroni correction (see Table 3). More specifically, the Cyberbully-victim group showed the highest levels of depressive symptoms, and the lowest levels of subjective well-being, and family support. Higher levels of anxiety symptoms were observed for both the cyber-victim and the cyberbully-victim groups. There were no significant differences between the three cyberbullying roles (i.e., cyberbully, cyber-victim, and cyberbully-victim) in social support from friends and teachers. Effect sizes were small for all the analyzed variables except depressive symptoms, which showed a medium effect size. 

### 3.3. Cyberbullying Roles and Psychological Well-Being: The Potential Mediational Role of Perceived Social Support

In order to include cyberbullying roles as independent variables in the mediation models, three dummy variables were created: Cyberbully (No (0) = 1687; Yes (1) = 13), Cyber-victim (No (0) = 1509; Yes (1) = 191), and Cyberbully-victim (No (0) = 1673; Yes (1) = 27). Three independent models were initially estimated, examining the effects of cyberbullying roles and social support variables on depression (RMSEA = 0.04, CFI = 0.93, SRMR = 0.04), anxiety (RMSEA = 0.04, CFI = 0.95, SRMR = 0.03), and subjective well-being (RMSEA = 0.04, CFI = 0.96, SRMR = 0.03), respectively. Because similar results were observed across models, and considering the covariance between depressive symptoms, anxiety symptoms, and subjective well-being, the final model tested the effects of cyberbullying roles and perceived social support on all three psychological well-being outcomes simultaneously. 

**Figure 1:**
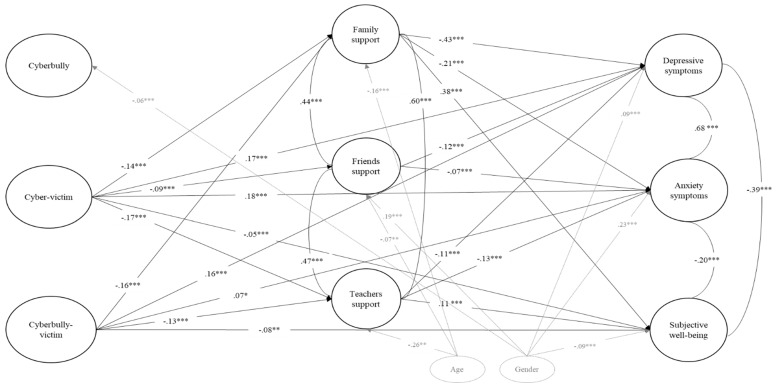
Mediation model of cyberbullying roles on psychological well-being through perceived social support. Root-Mean-Square Error of Approximation (RMSEA) = 0.03; Comparative Fit Index (CFI) = 0.92; Standardize Root Square Residual (SRMR) = 0.04. The model shows the standardized estimates for direct effects, covariance between mediators and dependent variables, and controlled effects for age and gender (in grey). Only statistically significant relationships are shown in the figure. * *p* < 0.05. ** *p* < 0.01. *** *p* < 0.001.

As can be seen in Figure 1, model fit indices ranged from acceptable (CFI = 0.92) to good (RMSEA = 0.03; SRMR = 0.04). Regarding direct effects, both Cyber-victim and Cyberbully-victim roles showed a positive and significant association with depressive and anxiety symptoms and negative associations with subjective well-being. Similarly, the Cyber-victim group was significantly and negatively related with all perceived types of social support (i.e., from family, friends, and teachers), whereas the Cyberbully-victim showed negative significant associations with family and teachers support. Finally, family, friends’, and teachers’ support showed negative associations with both depressive and anxiety symptoms. Only family and teachers’ support showed a positive and significant association with subjective well-being. 

Of note, being a Cyberbully did not show direct associations with any of the analyzed variables. Therefore, mediation effects were only examined for Cyber-victim and Cyberbully-victim roles (see Table 4). Indirect effects, although small, showed that the association between the Cyber-victim and Cyberbully-victim groups with all three psychological well-being outcomes were partially mediated by family and teachers support, except for Cyberbully-victim on subjective well-being through teacher support, which was non-significant. More specifically, for Cyber-victims and Cyberbully-victims, higher levels of family and teachers’ support reduce the probability of showing depressive and anxiety symptoms, whereas higher levels of family support increase the probability of reporting higher levels of subjective well-being. 

## 4. Discussion

In this study, we examined the relations between cyberbullying and its different roles with symptoms of depression, anxiety, and levels of subjective well-being. In addition, and in order to identify potential variables that may mediate these associations, we tested whether and to what extent youths’ perceived social support from family, friends, and teachers mediate the association between different cyberbullying roles and the three studied psychological well-being outcomes. We conclude from our results that the Cyberbully-victim group has the highest levels of depressive symptoms, and the lowest levels of subjective well-being and family support. We also observed higher levels of anxiety symptoms in both the Cyber-victim and the Cyberbully-victim roles. Moreover, we conclude that some types of social support seem protective in the way that it mediates the relationship between cyberbullying and psychological well-being. More specifically, perceived social support from family and from teachers reduce the probability of depressive and anxiety symptoms, and higher levels of social support from the family increase the probability of higher levels of subjective well-being among youths being a victim of cyberbullying (i.e., Cyber-victim) and being both a perpetrator and a victim of cyber bullying (i.e., Cyberbully-victim). 

In current study, the prevalence of involvement in cyberbullying was very low according to the self-reports of the young adolescents in this study. This is of course a positive sign since it indicates low levels of victimization and perpetration of cyberbullying. In comparison to other studies, our results are in line with others who show relative low levels of prevalence’s of involvement in cyberbullying, but not with those who report much higher numbers (for meta-review of prevalence, see Reference [51]). Due to the lack of consensus regarding the definition of cyberbullying and hence difficulties in the operationalization of the concept, multiple instruments are used in the field to estimate cyberbullying [52], making comparisons across studies problematic. In our study, we used a single item to estimate involvement in cyberbullying. Even though the use of such a single item has been argued to be both an economical, valid, and reliable estimate of bullying [42], it also comes with some limitations that perhaps explain the low prevalence of cyberbullying involvement reported in the current study. By using the word bullying/bully/victim in the questions, there is always a risk of biased perception or stigma [53]. In addition, single-item rather than multiple-item scales also run the risk of missing the complexity of a phenomenon such as cyberbullying, hence rendering lower prevalence than multiple-item scales [51,54]. Also, relying on self-report as the single strategy to collect information about bullying behavior may be problematic and some argue for the use of multiple reporters and methods to assess bullying [51,55]. As an example, peer and teacher nominations can be important additions to youth self-reports, as previous studies indicate higher agreement between peers and teachers in identifying both bullies and victims, than in relation to youth self-report [55]. Hence, future studies could benefit from combining multiple sources of reports when estimating cyberbullying involvement. 

In line with previous cross-sectional studies, our results show how involvement in bullying is related to both depressive and anxiety symptoms, as well as to lower levels of subjective well-being [5,12,13,14,15,16,17,18,19,20]. These results are consistent with previous research indicating the harm that cyberbullying may have on youths irrespectively of being a bully or a victim [5]. In addition, our results show that Cyberbully-victims reported the highest levels of depressive symptoms, as well as the lowest levels of subjective well-being. This is also in line with previous research indicating that this group of children may be at an extra risk for adverse outcomes [24,25]. Also, Cyberbully-victims also showed the lowest levels of perceived family support. This is important knowledge, since there is a lack of research on this particular group in general, and more specifically, in relation to sources of perceived support. 

Expanding on previous research, our results show that higher levels of family and teacher social support are related to lower levels of depressive and anxiety symptoms among both Cyber-victims and Cyberbully-victims. Similar results have been found in relation to traditional non-cyber bullying, where parent and teacher support has been found to buffer against internalizing problems [27]. More specifically, using a longitudinal design, cyber-victimized adolescents who sought support from family reported lower levels of depression one year later [39]. In addition to this, our results expand on this knowledge, showing that positive social support can be beneficial, not only for Cyber-victims but also for Cyberbully-victims. There are numbers of ways that family and teacher support might translate into less negative outcomes for youth involved in cyberbullying. Parents and teachers could offer emotional support to handle the distress of being involved in cyberbullying [27]. The adult perspective in offering support is important, since others argue that peers might not see cyberbullying as problematic as adults and hence, not offer the support needed by youths involved in cyberbullying [35]. Also, parents and teachers might be able to intervene earlier to decrease or even stop the bullying. The potential role of parents as protectors has also been shown by others in that parental support has been related to less cyber-victimization one year later [32]. Our results add important new knowledge to the field by identifying social support factors that might mitigate the potential negative consequences of cyberbullying. However, it is important to note that the effect sizes were small. 

In contrast to family and teacher support, perceived support from friends was not shown to buffer against psychological impairment from involvement in cyberbullying, regardless of being a Cyber-victim, Cyberbully, or Cyberbully-victim. These results are partially in line with previous studies on traditional non-cyberbullying; however, it is important to note that previous studies on social support as a mitigating factor have yielded mixed results. Our results are in line with those previous studies in which perceived social support from friends was not shown to protect children involved in traditional bullying from adverse outcomes such as depression [9], or internalizing problems [6]. However, in other studies on traditional bullying, perceived support from friends was demonstrated to mitigate the impact of bullying on the quality of lives of victims [32], as well as on anxiety/depression [7]. In relation to cyberbullying, our results contradict one of the few studies that have examined the issue and in which seeking support from friends had a buffering effect on depressive symptoms among cyber-victims [39]. However, our study expands this knowledge since they in their study, unlike we in ours, did not include Cyber-bullies nor Cyberbully-victim roles. The lack of studies, and the mixed results within the field, emphasizes the importance of more studies on friends’ support and its potential buffering function. This might be of particular importance since other studies indicate that seeking help or telling a friend is a commonly used coping strategy by youth who experience cyber-victimization [35]. However, our results indicate that seeking support from friends might not be the best way of coping with cyberbullying. One explanation could be that friends do not see cyberbullying as problematic as adults do [35], and hence do not offer the support that cyber-involved youths need. It could also be the case that friends are not able to help stop the bullying in the same way as parents or teachers could, since they have other resources to intervene in the situation. Regardless, since prior research indicates that youths commonly use and recommend others to use friends as support when experience cyberbullying [37,56], the role of friends’ support should be an important question for future studies. 

In relation to the current studies results on the potential positive aspects of social support, there seems to be a positive bias towards adult support for cyberbullying-involved youths, i.e., youths who are involved in bullying and perceive higher levels of support from adults (i.e., family or teachers) may have less adverse outcomes than those who report less. Similar results on the positive aspects of parent/family and teacher support for cyberbullied children have been found in previous research [32,39]. These results are important, especially since studies indicate that adolescents generally do not tell a teacher or a parent when they are cyberbullied [33,35,56,57]. In fact, adolescents report that telling a teacher is rather ineffective [33] and that seeking support from a parent might be related to the loss of privileges such as free and unsupervised use of the Internet [57]. Hence, as argued by other scholars, adults should take a proactive approach in taking responsibility for building a supportive relationship with the youth [5,58]. 

Notwithstanding the prior contributions, the results of the present study should be interpreted in the light of the following limitations. First, the reliance on self-reports for all measures could have inflated some results due to shared method variance. Future research would benefit from using multiple sources of information, such as teacher and parent reports. However, a strength of this study is its use of well-validated scales to measure the majority of the included concepts. However, subjective well-being is a self-developed item, but can be argued to have high face-validity, although there were no other ways to validate this item. Second, the cross-sectional design did not allow for testing prospective associations and, therefore, conclusions of direction or stability of effects could not be established. Hence, future research should apply a longitudinal design in order to understand how cyberbullying may affect adolescents´ psychological well-being over time, and also, which role different sources of social support might play in such relations. This is of great importance since it is not clear from previous research if aspects of psychological well-being such as depressive and anxiety symptoms, as well as perceived support, are antecedents or consequences of involvement in cyberbullying [5]. In fact, it could be that there is a bidirectional link between studied variables. For example, psychological problems such as depressive symptoms could result in less social skills, and a tendency for youths to withdraw themselves from others, making them less attractive to peers, hence increasing the likelihood of becoming a victim of bullying [15]. Also, social support may work as a protective factor for children not to become involved in cyberbullying. In fact, youths may be interpersonally at risk (having fewer friends or being rejected by peer) for future victimization [59]. Also, family support has been proven to decrease the risk for cyber-victimization one year later [32]. Hence, more research should use a longitudinal approach to further clarify the potential causal relationships between psychological well-being, social support, and involvement in cyberbullying. Third, although the current study included a relatively large sample, the prevalence rates for both cyberbullying and cyber-victimization were relatively low, leading to small groups when testing the cyberbullying roles. Related to this, the small size of the groups did not allow for further tests by gender, an issue that should be addressed in future research. Hence, research using larger samples is needed to increase our understanding of how factors such as gender may moderate the association between cyberbullying roles, psychological well-being, and different sources of social support. This is of great importance since prior research indicates that seeking support may be more beneficial for girls compared to boys [5], and also that different sources of support might be of different importance for bullied girls and boys [6]. Lastly, the effect sizes for the indirect effects were small. Since we excluded the suicide item from the depressive symptoms scale due to ethical considerations, i.e., the setting for the answering of questionnaire and the age of respondents, this could have potentially attenuated the strength of the statistical relationships. Hence, including suicide ideation in the depressive symptoms scale could potentially have helped to differentiate between the groups and outcomes. 

## 5. Conclusions

In conclusion, the current study suggest that youths involved in cyberbullying, as victims, bullies, and bully-victims, are at increased risk for anxiety and depressive symptoms, as well as less well-being in general. Since cyberbullying has been linked to adverse outcomes in adolescence, it is important that teachers and other practitioners have a good understanding of how children’s different involvement in bullying relates with psychosocial well-being outcomes, and which variables that may impact that relationship. As observed in previous research, both emotional and instrumental perceived social support could serve as important resources when youths experience bullying in its different roles (e.g., References [9,32]). In this regard, perceived social support, particularly from family and teachers at school, may ameliorate the potential link between cyberbullying and several distress outcomes at the psychosocial well-being level. Hence, prevention and intervention programs of cyberbullying could benefit from including specific strategies leading to an increase in both seeking and perception of functional social support from relevant figures in adolescence (i.e., family and teachers).

## Figures and Tables

**Table 1:** Descriptive statistics for the main study variables with tests for gender differences.

		Total Sample	Boys	Girls	
	Min–Max	Mean (SD)	Mean (SD)	Mean (SD)	*t*
Cyberbullying	1.00–5.00	1.04 (0.28)	1.05 (0.35)	1.02 (0.16)	2.45 *
Cyber-victimization	1.00–5.00	1.18 (0.57)	1.19 (0.60)	1.18 (0.53)	0.31
Depressive symptoms	1.00–3.00	1.36 (0.33)	1.36 (0.61)	1.35 (0.34)	0.07
Anxiety symptoms	1.00–4.00	1.73 (0.54)	1.63 (0.50)	1.84 (0.55)	−8.53 ***
Subjective well-being	1.00–4.00	3.45 (0.79)	3.51 (0.76)	3.38 (0.82)	3.38 ***
Family support	1.00–4.00	3.57 (0.55)	3.58 (0.52)	3.55 (0.58)	1.38
Friends support	1.00–4.00	3.45 (0.64)	3.35 (0.66)	3.56 (0.60)	−7.07 ***
Teachers support	1.00–4.00	3.27 (0.74)	3.29 (0.74)	3.25 (0.75)	0.87

Note. Min = Minimum score; Max = Maximum score; SD = Standard deviation. * *p* < 0.05. ** *p* < 0.01. *** *p* < 0.001.

**Table 2:** Zero-order correlations between the main study variables.

	1	2	3	4	5	6	7	8
1. Cyberbullying	-							
2. Cyber-victimization	0.30 ***	-						
3. Depressive symptoms	0.18 ***	0.30 ***	-					
4. Anxiety symptoms	0.04	0.23 ***	0.57 ***	-				
5. Subjective well-being	−0.07 **	−0.16 ***	−0.49 ***	−0.33 ***	-			
6. Family support	−0.14 ***	−0.18 ***	−0.45 ***	−0.28 ***	0.40 ***	-		
7. Friends support	−0.05 *	−0.10 ***	−0.31 ***	−0.17 ***	0.23 ***	0.40 ***	-	
8. Teachers support	−0.11 ***	−0.20 ***	−0.41 ***	−0.28 ***	0.36 ***	0.55 ***	0.42 ***	-

* *p* < 0.05. ** *p* < 0.01. *** *p* < 0.001.

**Table 3:** Comparisons between cyberbullying roles on psychological well-being and social support variables.

	No Cyberbully/Victim (n = 1469)	Cyberbully (n = 13)	Cyber-Victim (n = 191)	Cyberbully-Victim (n = 27)		
	Mean (SD)	Mean (SD)	Mean (SD)	Mean (SD)	F	ƞ²
Depressive symptoms	1.32 (0.30)_a_	1.50 (0.34)_ab_	1.55 (0.38)_b_	1.86 (0.48)_c_	57.62 *	0.09
Anxiety symptoms	1.68 (0.49)_a_	1.58 (0.52)_a_	2.06 (0.64)_b_	2.11 (0.83)_b_	35.32 *	0.06
Well-being	3.50 (0.76)_c_	3.54 (0.78)_bc_	3.20 (0.85)_b_	2.56 (1.01)_a_	20.61 *	0.04
Family support	3.61 (0.51)_c_	3.31 (0.85)_bc_	3.38 (0.68)_b_	3.00 (0.80)_a_	21.42 *	0.04
Friends support	3.47 (0.63)_b_	3.21 (0.71)_ab_	3.30 (0.70)_a_	3.36 (0.55)_ab_	4.82 *	0.01
Teachers support	3.32 (0.72)_b_	3.16 (0.78)_ab_	2.93 (0.80)_a_	2.58 (0.93)_a_	25.16 *	0.04

Note. ƞ² = partial effect size statistic. Means with different subscripts (a, b, c) were significantly different (*p* < 0.05) in post hoc pairwise comparisons (subscript a represents the lowest score/s in the analyzed variable). * Significant value after applying the Bonferroni correction (*p* < 0.008).

**Table 4:** Standardized indirect effects of cyberbullying roles (Cyber-victim and Cyberbully-Victim) on psychological well-being outcomes through family, friends’, and teachers’ support.

	β	95% CI
Cyber-victim/Family/Depress	0.06 ***	0.03, 0.09
Cyber-victim/Friends/ Depress	0.01 *	0.00, 0.02
Cyber-victim/Teacher/ Depress	0.02 *	0.01, 0.03
Cyber-victim/Family/Anxiety	0.03 **	0.01, 0.05
Cyber-victim/Friends/Anxiety	0.01	0.00, 0.01
Cyber-victim/Teacher/Anxiety	0.02 *	0.01, 0.03
Cyber-victim/Family/Well being	−0.05 ***	−0.08, −0.03
Cyber-victim/Friends/Well being	−0.01	−0.01, 0.00
Cyber-victim/Teacher/Well being	−0.02 **	−0.03, −0.01
Cyberbully-victim/Family/ Depress	0.07 ***	0.04, 0.10
Cyberbully-victim/Friends/ Depress	0.00	0.00, 0.01
Cyberbully-victim/Teacher/ Depress	0.02 *	0.01, 00.02
Cyberbully-victim/Family/Anxiety	0.03 **	0.02, 0.05
Cyberbully-victim/Friends/Anxiety	0.00	−0.00, 0.00
Cyberbully-victim/Teacher/Anxiety	0.02 *	0.01, 0.03
Cyberbully-victim/Family/Well being	−0.06 ***	−0.09, −0.03
Cyberbully-victim/Friends/Well being	−0.00	−0.00, 0.01
Cyberbully-victim/Teacher/Well being	−0.01	−0.00, 0.01

Note. CI = Confidence interval. * *p* < 0.05. ** *p* < 0.001. *** *p* < 0.001.
